# Effect of window-to-wall-area ratio on thermal performance of building wall materials in Elazığ, Turkey

**DOI:** 10.1371/journal.pone.0237797

**Published:** 2020-09-01

**Authors:** Meral Ozel, Cihan Ozel

**Affiliations:** Department of Mechanical Engineering, Firat University, Elazığ, Turkey; Universite des Sciences et de la Technologie Houari Boumediene, ALGERIA

## Abstract

In this study, the effect of glazing-to-total-wall-area ratio on the thermal performance of different wall materials is numerically investigated in terms of heat transmission load. The investigation was performed for a South-facing wall in Elazığ, Turkey. The heat transmission load through walls and windows are determined separately for summer and winter climate conditions. In this analysis, the frame area of the window is not considered. Therefore, whereas the glazing area on uninsulated and insulated walls is increased from 0% to 100%, the heat gain and losses are calculated separately according to the glazing type. The transmission loads through the wall are determined by an implicit finite difference procedure under steady periodic conditions. Concrete, briquettes, bricks, and autoclaved aerated concrete are selected as structure materials. Results show that in the uninsulated wall, the wall material affected the glazing area, whereas in the insulated wall, the effect of wall material on glazing area is insignificant.

## 1. Introduction

Solar radiation is responsible for 40%–70% of cooling load in buildings. Minimizing solar heat gain from walls and windows can significantly reduce energy consumption for air-conditioning [1]. Heat transfer from transparent surfaces differ significantly from that through opaque surfaces. Owing to their transparency, glazed facades transmit sunlight directly to buildings [2]. Windows are often the weakest factor in buildings for energy reduction. Approximately one-third of energy loss from a typical house is from windows. However, windows are essential in architectural applications because they improve the appearance of buildings and enable daylight penetration [3]. Furthermore, building walls are thermal storage elements, and energy stored in the walls during the day can be used for heating the building at night. Meanwhile, using thermal insulation improves the thermal performance of building wall elements and reduces heat transmission load by increasing the thermal resistance.

Many studies have been performed regarding the thermal performance of building elements [4–7], in particular, wall and roof elements. However, the effect of window-to-wall-area ratio on thermal behavior has been studied by only a few researchers [8–22], in which different wall and windows were investigated under different climatic conditions.

Although many studies regarding the thermal performance of walls containing windows exist, none of them reveal the effect of glazing area on the thermal performance of building structure materials. The effect of glazing area on the thermal performance of buildings has been previously investigated by Ozel [22], in which wall orientations and glazing were considered. In a previous study, different building materials were not considered while wall orientations were investigated for all months of the year. The main purpose of the present study is to examine the effect of wall material on the glazing area in terms of heat transmission. Therefore, in this study, the effects of different structure materials, such as concrete, briquettes, bricks, and autoclaved aerated concrete (AAC) on glazing area were investigated for insulated and uninsulated walls in terms of heat gain and losses. The investigation was performed for two types of glazing in the South façade of a building in the summer and winter climatic conditions of Elazığ, Turkey.

## 2. Mathematical formulation

### 2.1. Heat transmission through wall

**Fig 1:**
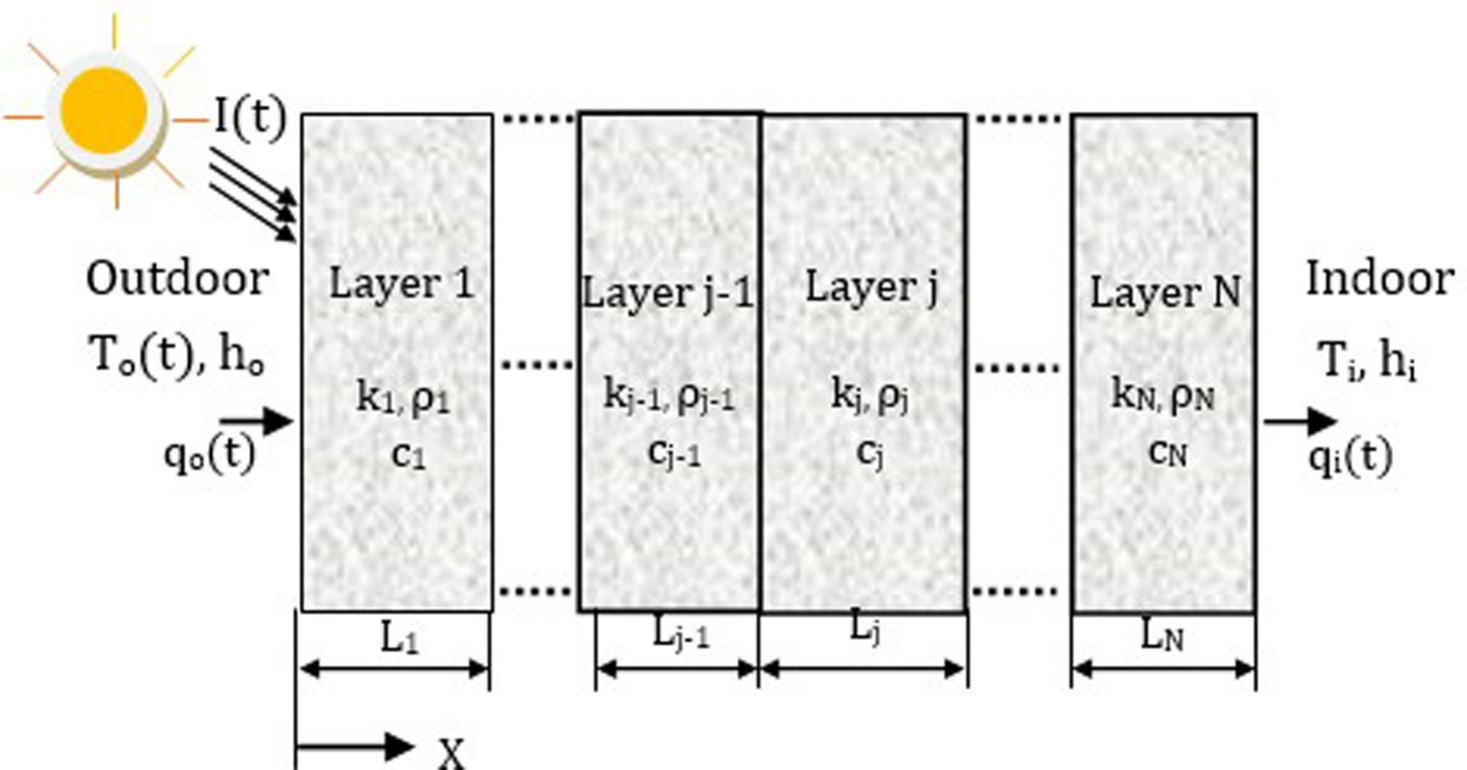
A multilayer wall structure.

Heat transmission through a multilayer wall, as shown schematically in Fig 1, may be calculated by solving a transient one-dimensional heat conduction equation as follows:

$$\rho_{j}c_{j}\left(\frac{\partial T_{j}}{\partial t}\right)=k_{j}\left(\frac{\partial^{2}T_{j}}{\partial x^{2}}\right),\;j=1,2,\ldots N$$ (1)

where *ρ*_*j*_, *c*_*j*_, and *k*_*j*_ are the density, specific heat, and thermal conductivity of the *j* th layer, respectively. The thermal conduction at the interfaces between the layers may be expressed by the following equations [23, 24]:

$$T_{j}=T_{j+1},\;j=1,2,\ldots (N-1)$$ (2)

$$k_{j}\left(\frac{\partial T_{j}}{\partial x}\right)=k_{j+1}\left(\frac{\partial T_{j+1}}{\partial x}\right),\;j=1,2,\ldots ,(N-1)$$ (3)

An arbitrary uniform temperature as an initial condition was assumed. The convection boundary conditions on both surfaces of wall are as follows [23, 24]:

$$-k_{N}\frac{\partial T(L,t)}{\partial x}=h_{i}(T_{x=L}-T_{i})$$ (4)

$$-k_{1}\frac{\partial T(0,t)}{\partial x}=h_{0}(T_{sa}(t)-T_{x=0})$$ (5)

where *T*_*i*_ is the constant indoor air temperature; *h*_*i*_ and *h*_*o*_ are heat-transfer coefficients at the inner and outer surfaces of the wall, respectively. *T*_*sa*_ is the sol-air temperature that varies throughout the day and is expressed for vertical surfaces as follows [25]:

$$T_{sa}=T_{o}+\frac{\alpha_{o}I_{}}{h_{o}}$$ (6)

where *T*_*o*_ and *I* are the outdoor air temperature and the total solar radiation that vary throughout the day, respectively; *α*_o_ is the solar absorbtivity of the outer surface of the wall surface. *I* is calculated for vertical surfaces as follows [26]:

$$I_{}=R_{b}I_{d}+(I_{y}+I_{a}\rho_{y})/2$$ (7)

where *I*_*d*_, *I*_*y*_, and *I*_*a*_ are the instant direct, diffuse, and total solar radiations on the horizontal surface; *ρ*_*y*_ is the ground reflectance. Parameter *R*_*b*_ is expressed as follows:

$$R_{b}=\frac{\cos\theta }{\cos\theta_{z}}$$ (8)

where *θ*and *θ*_*z*_ are the incident and zenith angles, respectively [26].

#### 2.1.1. Numerical solution procedure

**Fig 2:**
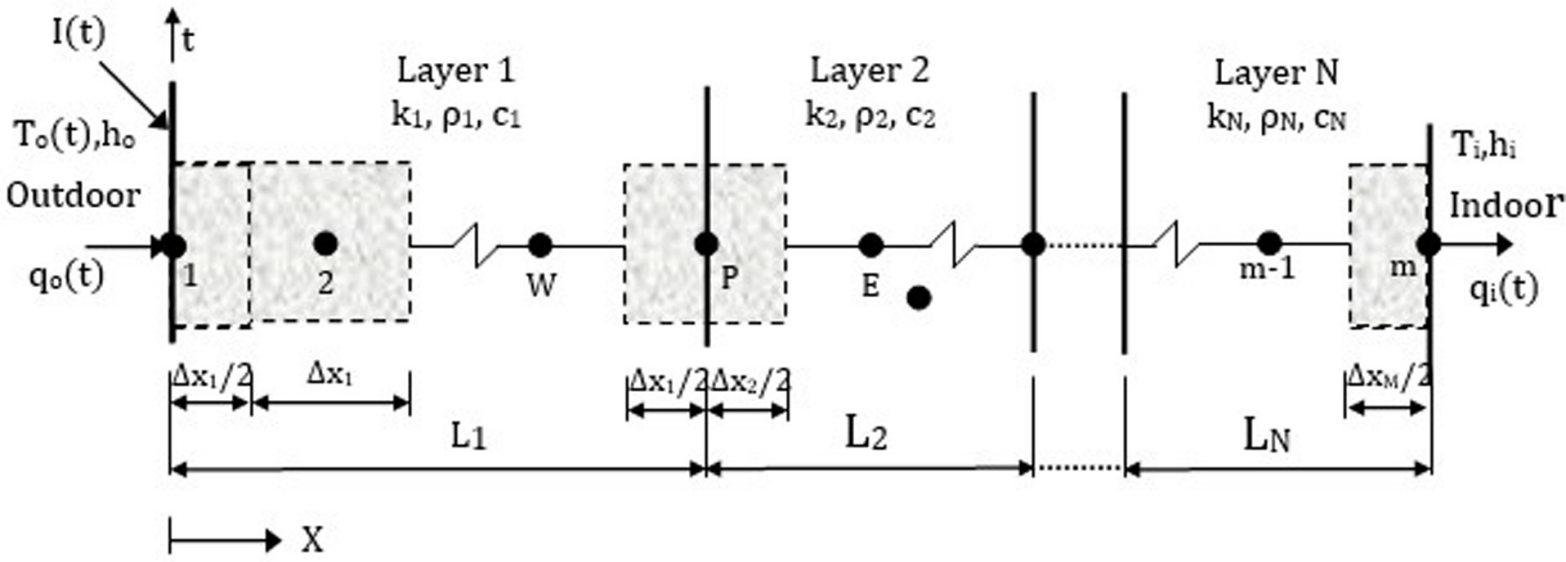
The multilayer wall showing grid nodes.

Differential equation and boundary conditions were numerically solved by implicit finite difference method for a wall with N layers, discretized as shown in Fig 2. Finite-difference equations were obtained for the boundary node on the outside, interior nodes in the layers, interface nodes between layers, and the boundary node on the inside, as follows, respectively [24]:

$$T_{1}^{n}+(2\lambda_{1}\beta_{o})T_{e}=[1+2\lambda_{1}(1+\beta_{o})]T_{1}^{n+1}-2\lambda_{1}T_{2}^{n+1}$$ (9)

$$T_{P}^{n}=-\lambda_{j}T_{W}^{n+1}+(1+2\lambda_{j})T_{P}^{n+1}-\lambda_{j}T_{E}^{n+1},$$ (10)

$$AT_{P}^{n}=-\frac{k_{j}}{\Delta x_{j}}T_{W}^{n+1}+(\frac{k_{j}}{\Delta x_{j}}+\frac{k_{j+1}}{\Delta x_{j+1}}+A)T_{P}^{n+1}-\frac{k_{j+1}}{\Delta x_{j+1}}T_{E}^{n+1},$$ (11)

$$T_{m}^{n}+(2\lambda_{N}\beta_{i})T_{i}=-2\lambda_{N}T_{m-1}^{n+1}+(1+2\lambda_{N}+2\lambda_{N}\beta_{i})T_{m}^{n+1}$$ (12)

$$\text{where}\;\beta_{o}=\frac{h_{o}\Delta x_{1}}{k_{1}},\;\beta_{i}=\frac{h_{i}\Delta x_{N}}{k_{N}},\;A=\frac{\left(\rho_{j}c_{j}\Delta x_{j}+\rho_{j+1}c_{j+1}\Delta x_{j+1}\right)}{2\Delta t},\;\lambda_{j}=\frac{k_{j}.\Delta t}{(\rho_{j}.c_{j}).{\left(\Delta x_{j}\right)}^{2}},\;(j=1,2,\ldots ,N).$$

The set of finite difference equations was solved using matrix functions in MATLAB, and the temperature distribution was obtained across the wall thickness at any time instant. To obtain a steady periodic solution, it was assumed that the sol-air temperature was repeated on consecutive days. The instant heat transmission load was determined as follows [24, 27]:

$$q_{iw}=h_{i}(T_{x=L}-T_{i})$$ (13)

Adequate accuracy of the numerical solution obtained by using implicit finite difference method has been previously obtained as Δx = 2.5 mm and Δt = 60 s according to the place and time steps, respectively [27]. Besides, it was shown that the numerical solution is in good agreement with the analytical solution [27].

### 2.2. Heat transmission through window

Heat transmission through the window glass comprised the total solar heat gain and heat transfer as a result of the temperature difference between the indoor and outdoor air. Heat flow through a single-glazed window can be calculated as follows [25]:

$$q_{isg}=F_{s}\tau_{D}I_{D}+\tau_{d}I_{d}+\tau_{R}I_{R}+(U/h_{o}).(F_{s}\alpha_{D}I_{D}+\alpha_{d}I_{d}+\alpha_{R}I_{R})+U(T_{o}-T_{i})$$ (14)

$$U=1/[(1/h_{i})+(1/h_{o})]$$ (15)

where *F*_*s*_ is the sunlit fraction and is assumed to be 1. Meanwhile, heat flow through a double-glazed window can be calculated as follows [28–30]:

$$\begin{array}{l} q_{idg}=F_{s}\tau_{(1,2)D}I_{D}+\tau_{(1,2)d}I_{d}+\tau_{(1,2)R}I_{R}+(U/h_{o}).(F_{s}\alpha_{(1of2)D}I_{D}+\alpha_{(1of2)d}I_{d}+\alpha_{(1of2)R}I_{R})+\\ U(1/h_{o}+1/h_{a}).(F_{s}\alpha_{(2of2)D}I_{D}+\alpha_{(2of2)d}I_{d}+\alpha_{(2of2)R}I_{R})+U(T_{o}-T_{i}) \end{array}$$ (16)

$$U=1/[(1/h_{i})+(1/h_{a})+(1/h_{o})]$$ (17)

where h_a_ is the air space heat transfer coefficient. The transmissivity, reflectivity, and absorptivity for a single glass are expressed as follows [25, 30, 31]:

$$\tau =\frac{{\left(1-r\right)}^{2}a}{1-r^{2}a^{2}}$$ (18)

$$\rho =r+r\frac{{\left(1-r\right)}^{2}a^{2}}{1-r^{2}a^{2}}$$ (19)

$$\alpha =1-r-\frac{{\left(1-r\right)}^{2}a}{1-ra}$$ (20)

where r is the component reflectivity; for an equal intensity of the components, r can be obtained using the Fresnel equation in the following form [25]:

$$r=\frac{1}{2}[\frac{{\text{sin}}^{2}(\theta -\theta ')}{{\text{sin}}^{2}(\theta +\theta ')}+\frac{{\text{tan}}^{2}(\theta -\theta ')}{{\text{tan}}^{2}(\theta +\theta ')}]$$ (21)

where *θ’* is the refraction angle and is expressed as [25]

$$\theta \text{'}={\text{sin}}^{-1}(\text{sin}\;\theta /n)$$ (22)

where n is the refraction index of glass. The absorption coefficient *a* is expressed as [25]

$$a=\exp[-KL/\sqrt{1-(\sin^{2}\;\theta /n^{2}})]$$ (23)

where *K* and *L* are the extinction coefficient and glass thickness, respectively. The properties for double glass are expressed as follows [25]:

$$\tau_{1,2}=\frac{\tau_{1}\tau_{2}}{1-\rho_{1}\rho_{2}}$$ (24)

$$\rho_{1,2}=\rho_{1}+\frac{\tau_{1}^{2}\rho_{2}}{1-\rho_{1}\rho_{2}}$$ (25)

$$\alpha_{1of2}=\frac{\left[1-(\rho_{1}+\tau_{1})][1-\rho_{2}(\rho_{1}+\tau_{1})\right]}{1-\rho_{1}\rho_{2}}$$ (26)

$$\alpha_{2of2}=\frac{[1-(\rho_{2}+\tau_{2})]\tau_{1}}{1-\rho_{1}\rho_{2}}$$ (27)

where subscripts 1 and 2 refer to the first and second sheets of glass considered, respectively.

### 2.3. Glazing-to-total-wall-area ratio

In this analysis, the frame area of the window was not considered. When only the glass area was considered, the glazing area in terms of percentage can be defined as follows [10]:

$$Glazing\;area\;(\%)=\frac{A_{g}}{A_{t}}.100=\frac{A_{g}}{A_{g}+A_{w}}.100$$ (28)

where *A*_*g*_ and *A*_*w*_ are the glass and wall areas, respectively. Whereas the glazing area on the wall was varied from 0% to 100% with an increment of 10%, the transmission loads were calculated separately for single and double glasses as follows:

$$Q_{i}=Q_{i}{}_{sg}.\frac{A_{g}}{A_{t}}+Q_{i}{}_{w}.(1-\frac{A_{g}}{A_{t}})$$ (29)

$$Q_{i}=Q_{i}{}_{dg}.\frac{A_{g}}{A_{t}}+Q_{i}{}_{w}.(1-\frac{A_{g}}{A_{t}})$$ (30)

where *Q*_*tw*_, *Q*_*isg*_, and *Q*_*idg*_ are the daily total loads for the wall, single-glazed window, and double-glazed window, respectively. To calculate these daily total loads, the instantaneous loads expressed by Eqs 13, 14 and 16 were integrated over 24 h periods.

## 3. Analyzed walls and windows

An investigation was performed for four different wall materials and two types of glazing (single and double glasses). Uninsulated and insulated wall structures were considered. The uninsulated wall comprised a 2 cm outer plaster, 20 cm structure material, and 2 cm inner plaster. Meanwhile, the insulated wall comprised a 2 cm outer plaster, 6 cm insulation layer, 20 cm structure material, and 2 cm inner plaster. Concrete, briquettes, bricks, and AAC as structure materials were selected since they are commonly used in building constructions in Turkey [33]. Table 1 lists the thermophysical properties such as thermal conductivity, density, specific heat and heat capacity of materials used in the wall structure [7]. It is seen that among examined structure materials, concrete wall has the highest thermal conductivity and highest heat capacity while AAC wall has the lowest thermal conductivity and lowest heat capacity.

**Table 1:** Thermophysical properties of building materials.

Material	k (W/mK)	ρ (kg/m^3^)	c (kJ/kgK)	ρ.c (kJ/m^3^K)
Concrete	1.731	2243	0.840	1884.12
Briquette	0.920	1600	0.840	1344.00
Brick	0.620	1800	0.840	1512.00
AAC	0.150	400	1.047	418.80
EPS	0.038	18	1.500	27.00
Plaster	0.720	1865	0.840	1566.60

For single glass, the glass thickness was 3 mm and for double glass, the thickness of each glass was 3 mm. The wall’s solar absorptivity was 0.8, whereas h_a_ was 5.56 W/m^2^K [26]. A heating absorbing glass was used. Therefore, K was 0.03 mm^-1^, whereas the refractive index (n) was 1.526 [21]. Varying outdoor air temperatures throughout the day were obtained from meteorology [32]. The indoor temperatures for January and July were assumed to be 20°C and 23°C, respectively; h_i_ and h_o_ were 9 and 22 W/m^2^K, respectively [33].

## 4. Results and discussion

**Fig 3:**
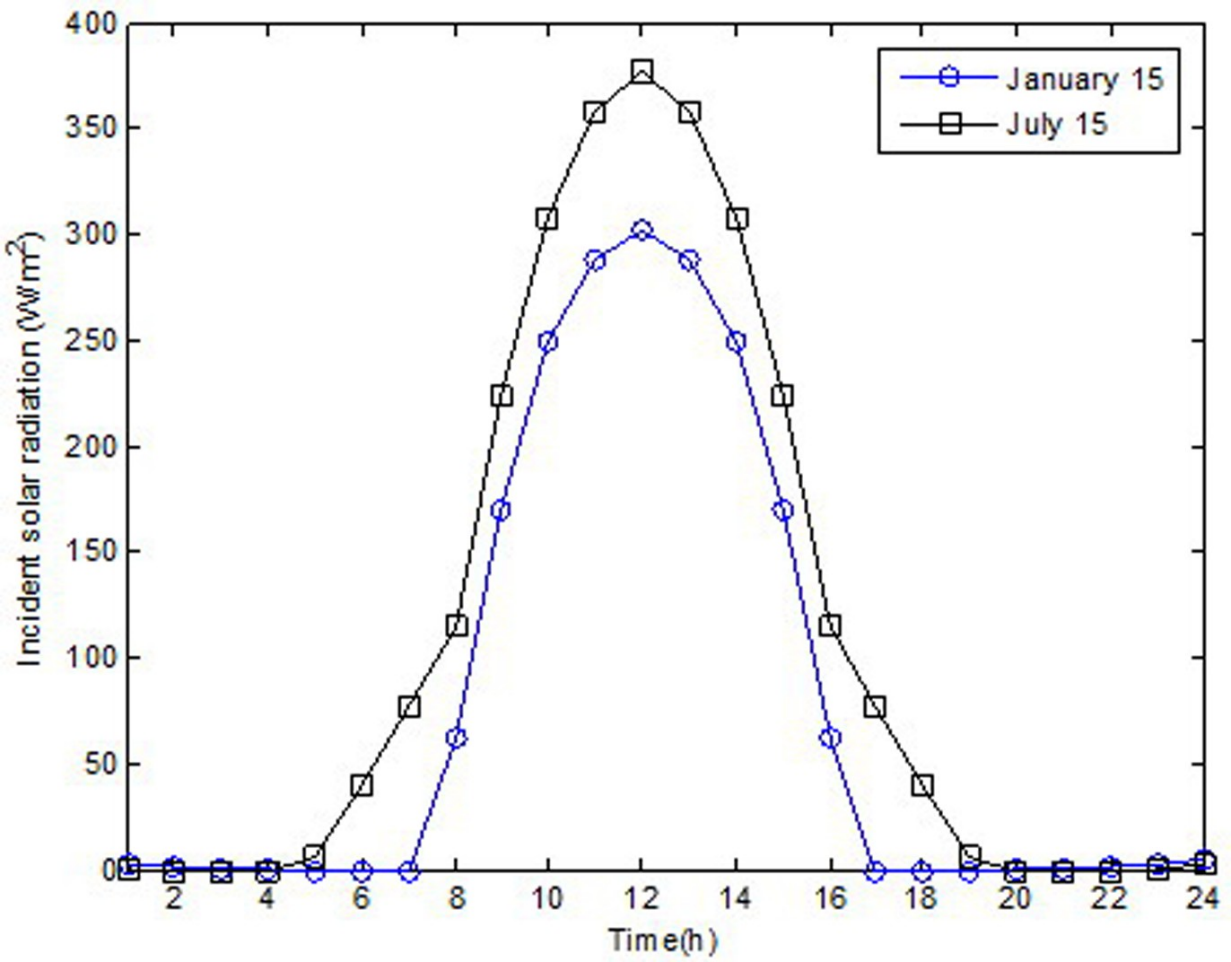
Hourly variation of incident solar radiation for January 15 and July 15.

**Fig 4:**
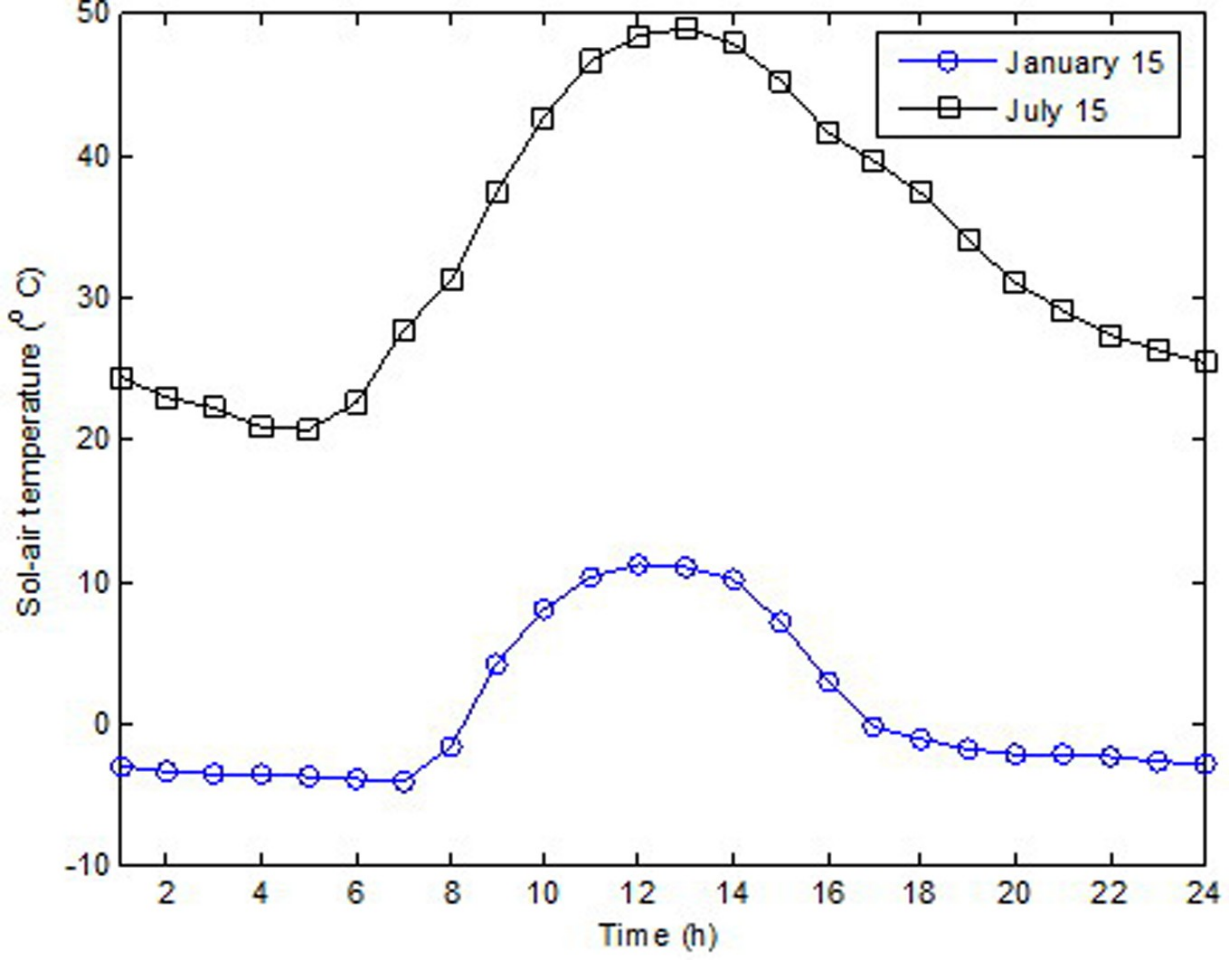
Hourly variation of sol-air temperature for January 15 and July 15.

In this study, effect of glazing area on building wall material was numerically investigated in terms of thermal performance. The investigation was performed for the south orientation in Elazığ, Turkey. Summers are hot and dry, while winters are cold and hard in Elazığ city (latitude: 38.40°N, longitude: 39.21°E) which is located in the Eastern Anatolia Region of Turkey. Furthermore, the investigation was performed on January 15 and July 15 to reflect typical winter and summer climatic conditions. Hence, the heat transmission loads according to increasing glazing area were calculated separately for single and double glasses. The hourly variation in incident solar radiation and sol-air temperature for January 15 and July 15 are shown in Figs 3 and 4, respectively. As shown in Fig 3, in the summer, the maximum solar radiation obtained was 376.71 W/m^2^; meanwhile, in winter, it was 301.77 W/m^2^ at 12:00. As show in Fig 4, in the winter, the maximum sol-air temperature was obtained at 12:00; meanwhile, in the summer, it occurred at 13:00.

**Fig 5:**
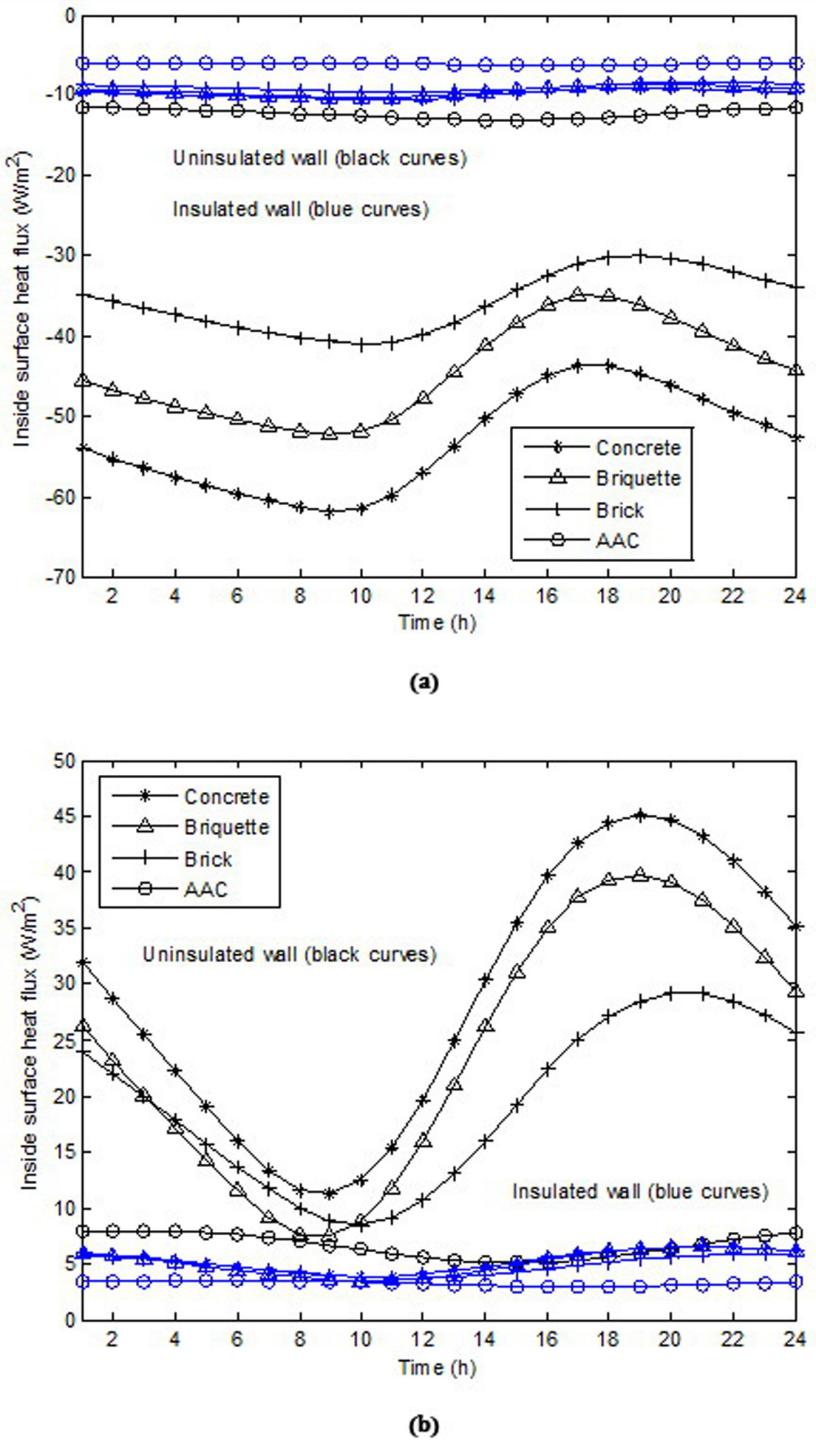
Hourly variation of inside surface heat fluxes of uninsulated and insulated walls constructed of concrete, briquette, brick and AAC: (a) for January 15 and (b) for July 15.

Fig 5A and 5B show the hourly variation in the inside surface heat fluxes of uninsulated and insulated walls constructed using concrete, briquettes, bricks, and AAC for January 15 and July 15, respectively. As shown, the heat gain and losses are the highest and lowest for the concrete wall and AAC wall, respectively. These results are consistent with those presented in [7, 33, 34], in which theoretical, numerical, and experimental models were used. The results show that in the winter, the peak heat fluxes on the uninsulated wall were 61.85, 52.36, 40.99, and 13.13 W/m^2^ for concrete, briquettes, bricks, and AAC, respectively; whereas in the summer, they were 45.04, 39.67, 29.14, and 8.036 W/m^2^, respectively. The amplitude of load fluctuation was determined from the difference between the maximum and minimum values of the inside surface heat flux. On the uninsulated wall in the winter, the amplitudes of the load fluctuation were 18.23, 17.40, 11.05, and 1.51 W/m^2^ for concrete, briquettes, bricks, and AAC walls, respectively. Meanwhile, in the summer, the corresponding values were 33.71, 32.11, 20.60, and 2.89 W/m^2^, respectively. The results show that the wall constructed of AAC yielded a smaller amplitude of load fluctuation and smaller peaks load in both the winter and summer. It was observed that, in the winter, the peak heat fluxes on the insulated wall were 10.59, 10.34, 9.62, and 6.22 W/m^2^ for concrete, briquettes, bricks, and AAC, respectively; whereas in the summer, they were 6.58, 6.59, 5.88, and 3.52 W/m^2^, respectively. The results show that the peak load reduced significantly when the wall was insulated. In the winter, this reduction was 82.87%, 80.25%, 76.53% and 52.62% for concrete, briquettes, bricks, and AAC, respectively. Similar conclusions were obtained in [7].

**Fig 6:**
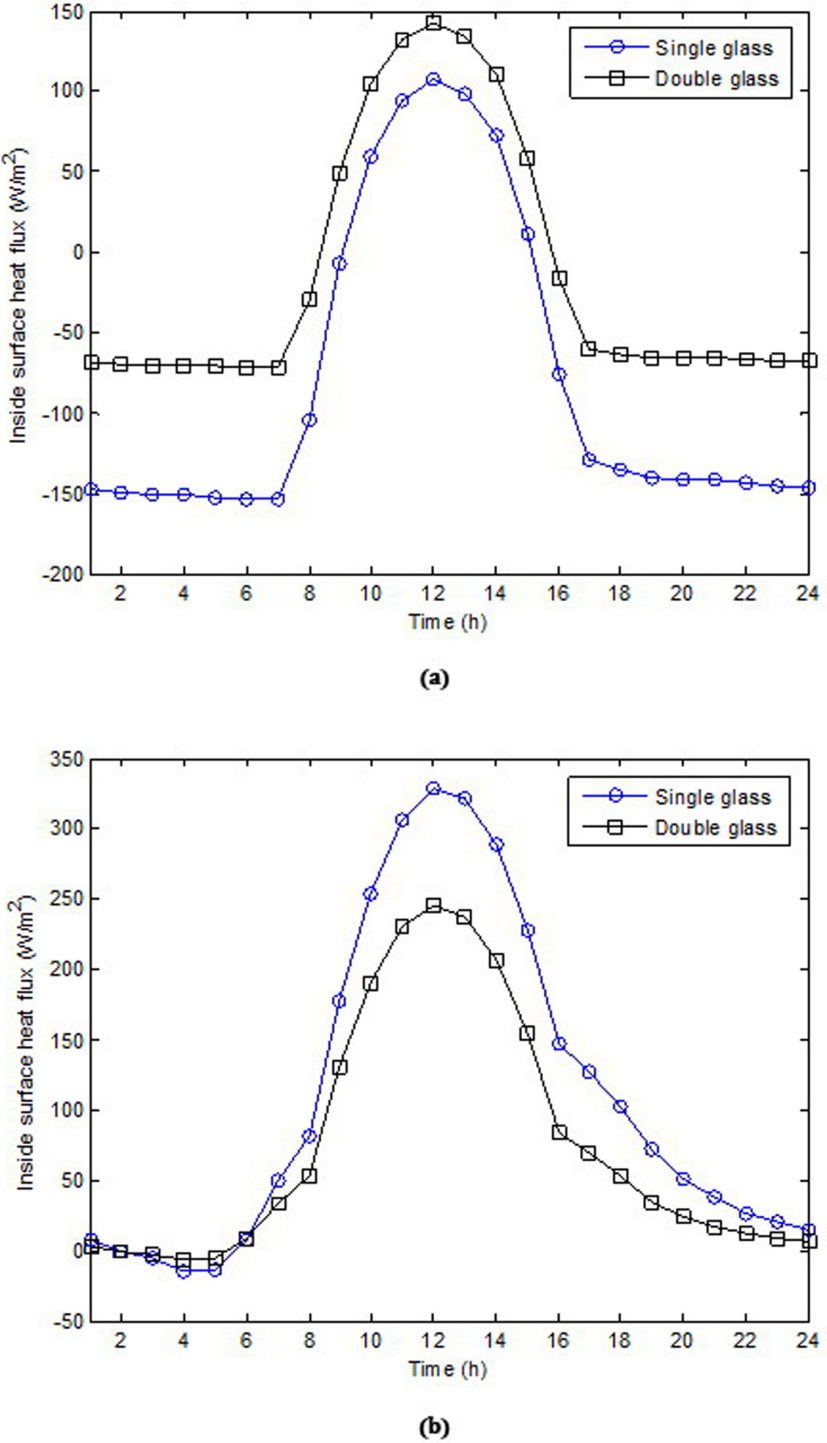
Hourly variation of inside surface heat flux through single glass and double glass: (a) for January 15 and (b) for July 15.

The daily fluctuations of the inside surface heat flux through single and double glasses for January 15 and July 15 are shown in Fig 6A and 6B, respectively. As shown, the maximum values of heat flux in the winter were 107.26 and 142.13 W/m^2^ for the single and double glasses, respectively. Meanwhile, the maximum values of heat flux in the summer were 327.78 and 245.84 W/m^2^ for single and double glasses, respectively. The results show that in the winter, the double-glazed windows increased heat gain compared with the single-gazed windows; whereas in the summer, the double-glazed windows reduced heat gain. It was discovered that in the summer, the reduction was 25%; whereas in the winter, the increase was 32.5%.

**Fig 7:**
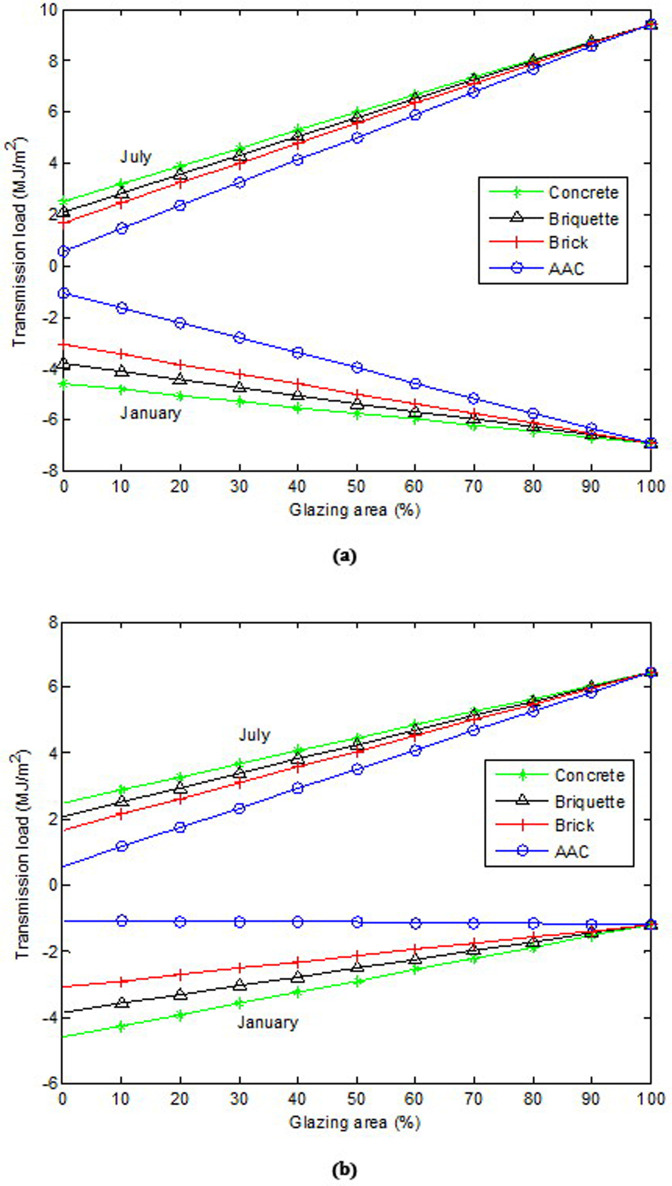
Variation of transmission loads versus glazing area according to different wall materials on uninsulated wall: (a) for single glazing and (b) for double glazing.

Fig 7A and 7B show the variation in transmission loads versus glazing area according to different wall materials on uninsulated single- and double-wall glazing, respectively. In the single-glazed uninsulated wall, as the glazing area increased, the heat gain and losses increased for all wall materials tested. The increase in the glazing area causes an additional heating and cooling load. This result is consistent with results of other studies obtained under different climatic conditions [8, 12]. It was clear that the increase in the cooling load was higher than the increase in the heating load. However, the effect of the glazing area on the heat loss of different wall materials was more prominent than that of heat gain. Furthermore, the glazing area of the concrete wall should be 60.4%, 39.6%, and 24.8% less than that of the AAC wall, brick wall, and briquette wall, respectively, in January; meanwhile, the corresponding numbers should be 21.7%, 10.6%, and 5.6%, respectively, in July. The results show that the glazing area of the AAC wall can be larger for both summer and winter conditions. In the double-glazed uninsulated wall, as the glazing area increased, the heat gain increased for all the wall materials tested. Meanwhile, the heat loss decreased in the bricks, briquettes, and concrete, whereas it increased slightly in AAC. This decrease was 61.5%, 69.1%, and 74.2% for the bricks, briquettes, and concrete, respectively. In July, the glazing area of the concrete wall should be 32.6%, 17.2%, and 9.5% less than that of the AAC wall, brick wall, and briquette wall, respectively. Meanwhile, the glazing area of the concrete wall in January was larger than those of the brick and briquette walls. The results show that in the winter, the glazing area of the brick wall should be 28.5% and 44.5% less than that of the briquette and concrete walls, respectively.

**Fig 8:**
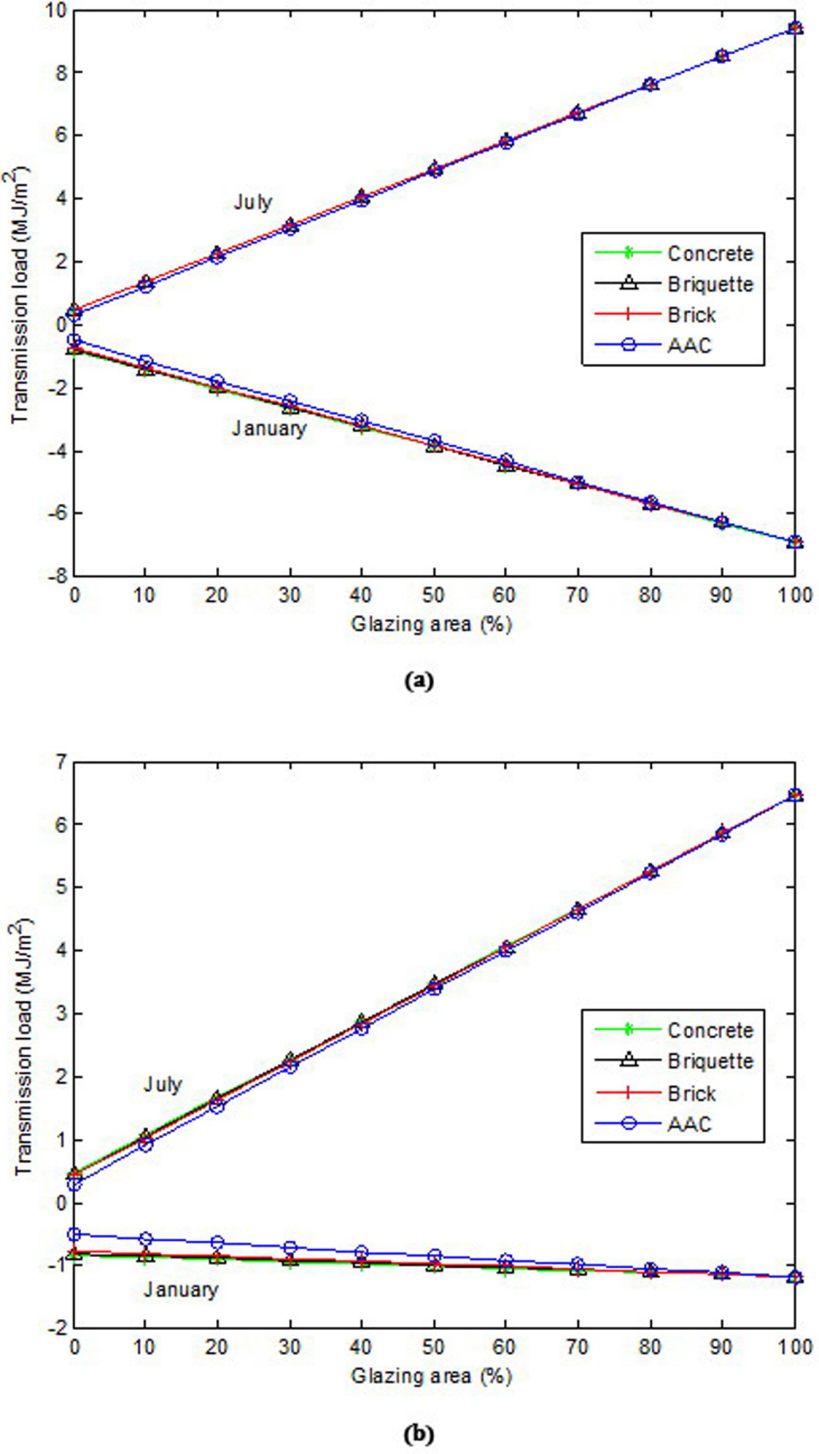
Variation of transmission loads versus glazing area according to different wall materials on insulated wall: (a) for single glazing and (b) for double glazing.

Fig 8A and 8B show the variation in transmission load versus glazing area according to different wall materials in single- and double-glazed insulated walls, respectively. In the single and double-glazed insulated walls, it was observed that as the glazing area increased, the heat gain and losses increased for all wall materials tested. For double glazing in the winter, the increase in heat loss was slight. Furthermore, the increase in heat loss and gain in double glazing was less than that in single glazing. The results show that in the uninsulated wall, the wall material affected the glazing area. However, in the insulated wall, the effect of wall material on the glazing area was insignificant.

## 5. Conclusion

The thermal performances of building external walls and window glasses were investigated in this study. The effect of glazing area on the thermal performance of building wall materials was numerically investigated in terms of heat gain and losses. The investigation was performed in the South orientation in the summer and winter climatic conditions of Elazığ, Turkey. Furthermore, the investigation was performed for four different wall materials and two different glazing types. The results indicated that in the uninsulated wall, the glazing area of ​​the AAC wall can be made larger for both summer and winter conditions. However, when the wall was insulated, the effect of building material on the glass area reduced significantly. In particular, it was discovered that the heat gain and losses of brick, briquette, and concrete walls did not change with increasing glass area.

## Abbreviations

a: absorption coefficient
A_g_: glass area (m^2^)
A_w_: wall area (m^2^)
c: specific heat (J/kg K)
F_s_: sunlit fraction of the window surface
h_i_: heat-transfer coefficient at the indoor surface of wall (W/m^2^ K)
h_o_: heat-transfer coefficient at the outdoor surface of wall (W/m^2^ K)
I_D_: incidence of direct radiation (W/m^2^) *I*_*R*_
I_d_: incidence of diffuse sky radiation (W/m^2^)
I_R_: incidence of solar radiation reflected (W/m^2^)
I: total solar radiations on the horizontal surface (W/m^2^)
k: thermal conductivity (W/m K)
K: extinction coefficient, (m^-1^)
L: glass thickness, (m)
*L*’: distance traveled by radiation, (m)
L_i_: insulation thickness (m)
n: index of refraction for glass
q_idg_: heat gain (or loss) ratio for a double-glazed window (W/m^2^)
q_isg_: heat gain (or loss) ratio for a single-glazed window (W/m^2^)
q_iw_: heat flux at indoor surface of the wall (W/m^2^)
Q_idg_: daily total load for a double-glazed window (MJ/m^2^)
Q_isg_: daily total load for a single-glazed window (MJ /m^2^)
Q_iw_: daily total load of the wall (MJ /m^2^)
r: reflectivity
t: time (s)
T_i_: indoor air temperature (°C)
T_o_: outdoor air temperature (°C)

## Greek letters

α_0_: solar absorptivity of outdoor surface of wall
α: absorptivity for a single sheet of glass
ρ_i_: density (kg/m^3^)
ρ: reflectivity for a single sheet of glass
θ: incidence angle (deg.)
θ_z_: zenith angle (deg.)
θ’: refraction angle, (deg.)
θ_ey_: incidence angle for diffuse sky radiation (deg.)
θ_ya_: incidence angle for reflected radiation (deg.)
τ: transmissivity for a single sheet of glass
